# The raccoon dog (*Nyctereutes procyonoides*) and the raccoon (*Procyon lotor*)—their role and impact of maintaining and transmitting zoonotic diseases in Austria, Central Europe

**DOI:** 10.1007/s00436-017-5405-2

**Published:** 2017-02-23

**Authors:** Tanja Duscher, Adnan Hodžić, Walter Glawischnig, Georg G. Duscher

**Affiliations:** 1 Research Institute of Wildlife Ecology, Department of Integrative Biology and Evolution, University of Veterinary Medicine Vienna, Vienna, Austria; 2 Institute of Parasitology, Department of Pathobiology, University of Veterinary Medicine Vienna, Veterinaerplatz 1, A-1210 Vienna, Austria; 3 Institute for Veterinary Disease Control, Austrian Agency for Health and Food Safety, Innsbruck, Austria

**Keywords:** *Alaria alata*, *Echinococcus multilocularis*, *Babesia* cf. *microti*, *Trichinella* spp., *Baylisascaris procyonis*, Neozoa

## Abstract

The neozoan species raccoon dog (*Nyctereutes procyonoides*) and raccoon (*Procyon lotor*) are widespread in Europe and potential vectors of many diseases that can threaten human and domestic animal health. Facing a further spread of these species, it is important to know about (i) pathogens imported and/or (ii) pathogens acquired in the new habitat. Thus, we investigated the parasite fauna of wild raccoon dogs and raccoons from Austria, at the edge of their new distribution range. The eight examined raccoons were nearly free of pathogens including *Baylisascaris procyonis*, and thus assumed to have a low epidemiological impact, so far. Out of ten raccoon dog specimens, we found one from western Austria to be infected with *Echinococcus multilocularis* and another three from the eastern wetland regions to harbour adults of *Alaria alata*. Furthermore, we detected *Babesia* cf. *microti* in five of eight raccoon dogs all over Austria but none of our samples were tested positive for *Trichinella* spp. Nevertheless, the raccoon dog seems to be a relevant host, at least for the zoonotic pathogens *E. multilocularis* and *A. alata,* and we suggest to further monitor the raccoon dogs parasite fauna.

## Introduction

Two of the most widespread non-indigenous wildlife species in Europe are the raccoon dog (*Nyctereutes procyonoides*) and the raccoon (*Procyon lotor*). Both species have been introduced to Europe in the twentieth century but their origin is completely the opposite, namely the USA for the raccoon and the Far East for the raccoon dog (Lutz 1984; Nowak 1973; Stubbe 1975). In the former decades, these new carnivore species expanded their range, increased in abundance, and even became the most common carnivore species in some parts of Europe (Kowalczyk 2014; Laurimaa et al. 2015). Consequently, there is a need to spend attention to these neozoa as additional wildlife reservoir of zoonotic diseases in Europe.

The most serious problem concerning the raccoon dog invasion seems to be the transmission of zoonotic diseases (Kauhala and Kowalczyk 2011). In Europe, the raccoon dog can be infected with a minimum of 32 helminth species of which 19 are zoonotic (Laurimaa et al. 2016). Its parasite fauna is similar to that of the indigenous red fox (*Vulpes vulpes*) (Al-Sabi et al. 2013; Bružinskaitė-Schmidhalter et al. 2012; Laurimaa et al. 2015; Thiess et al. 2001). The raccoon dog is assumed to be an important additional host species of *Echinococcus multilocularis* (Kauhala and Kowalczyk 2011; Schwarz et al. 2011; Tackmann et al. 2003) and moreover is highly susceptible for *Alaria alata* (Al-Sabi et al. 2013; Bružinskaitė-Schmidhalter et al. 2012; Laurimaa et al. 2016). The raccoon dog is also known to be an important reservoir host of *Trichinella* spp. (Bružinskaitė-Schmidhalter et al. 2012; Kauhala and Kowalczyk 2011; Thiess et al. 2001) and has been identified as important rabies vector in northeastern Europe (Holmala and Kauhala 2006; Singer et al. 2009). Due to broad oral vaccination campaigns, rabies is of less relevance in central Europe and is known to be eradicated in Austria since 2008 (Hirk et al. 2014). However, spill-over from neighbouring countries may still occur (Duscher et al. 2015). As the raccoon dog population is still growing and spreading, its role as a vector of this dangerous virus as well as of other zoonotic diseases may further increase in Europe (Kauhala and Kowalczyk 2011; Sutor et al. 2014).

While the raccoon is known to be the host of many helminth parasite species in its native range (e.g. Harkema and Miller 1964), previous studies from Germany, the country with the highest raccoon density in Europe, showed a remarkably low endoparasitic burden (e.g. Schwarz et al. 2015). The most prevalent parasites of raccoons found in recent studies were *Mesocestoides* spp. (Karamon et al. 2014; Schwarz et al. 2015). But the most serious zoonotic disease transmitted by raccoons in Europe seems to be the Larva migrans syndrome caused by *Baylisascaris procyonis*. The estimated prevalence of this parasite in European raccoon populations showed high geographical variations, ranging from 0 up to 71% (Al-Sabi et al. 2016; Gey 1998; Karamon et al. 2014; Schwarz et al. 2015; Winter et al. 2005). Concerning rabies, the raccoon is not considered to be a reservoir host in Europe, probably because of a low susceptibility for the relevant rabies virus variants (Vos et al. 2012). Among the piroplasmids, *Babesia* cf. *microti* was found in raccoons and raccoon dogs so far (Alvarado-Rybak et al. 2016; Han et al. 2010). This pathogen is frequently confirmed in foxes in Austria (Duscher et al. 2014), as well as in other regions of Europe sometimes infecting dogs (Camacho et al. 2004). This group comprises different clades and is also known as *Babesia microti*-like, *Babesia annae*, *Babesia* “Spanish dog isolate”, *Theileria annae* and *Babesia vulpes* (Baneth et al. 2015), but due to the lack of a valid description, it only can be informally named as the “microti group” (Harris 2016). Additionally, it has been stated that raccoons might get infected with at least three different *Babesia* species (Alvarado-Rybak et al. 2016).

The distribution and transmission of parasites depend on several environmental factors and thus differ across geographical regions (Dybing et al. 2013; Mackenstedt et al. 2015; Monello and Gompper 2011). So far, all studies concerning the parasite fauna of the raccoon dog as well as of the raccoon in Europe were conducted in the northeastern countries of its introduced range. Facing a range expansion of these neozoan species towards South Europe (Kauhala and Winter 2006; Winter 2006), further studies are needed to understand their role and impact of maintaining and transmitting zoonotic diseases in Europe’s diverse landscapes and climatic regions. Thus, we investigated the parasite fauna of wild raccoon dogs and raccoons from Austria, on the edge of both species’ geographic ranges. So far, the population densities in Austria do not reach high numbers but hunting bags are increasing (Duscher 2016). Therefore, sampling of statistical sufficient numbers to calculate parameters hardly seems possible. Nevertheless, any data of those species in the newly spread area is of great interest. It may give hints about (i) pathogens imported and/or (ii) pathogens acquired in the new habitat.

## Material and methods

Raccoons and raccoon dogs were obtained via regular hunting and catching events between February 2010 and January 2016 all over Austria under the restrictions of the Austrian game laws. Samples of in total eight raccoons and 13 raccoon dogs were obtained. Due to the small number of samples collected over 6 years, this is neither a longitudinal nor a cross-sectional study design. In some cases, only parts (e.g. intestines or tissue samples) were provided by the hunters. Either the whole animal or the intestines only, which were excised under high safety standards, were frozen at −80 °C for a minimum of 14 days to inactivate *E*. *multilocularis* oncosphaera. The intestines of seven raccoons and ten raccoon dogs were analysed by using the “shaking in a vessel” technique which was previously described (Duscher et al. 2005) (Table 1). Muscle tissues, at least 5 g if available (diaphragm or limb muscle), were analysed for *Trichinella* spp. by artificial digestion method at the National Reference Laboratory (Institute for Veterinary Disease Control, Austrian Agency for Health and Food Safety, Innsbruck, Austria). Spleens of four raccoons and eight raccoon dogs were analysed for piroplasmids (*Babesia/Theileria* spp.) and Anaplasmataceae (*Anaplasma* spp./*Ehrlichia canis* and *Candidatus* Neoehrlichia spp.) by using a nested and a single PCR, respectively, according to a protocol previously published (Duscher et al. 2013; Hodžić et al. 2015).

**Table 1:** Numbers of raccoons and raccoon dogs with the available material and the method used for analysis

Species/no.	Material available/method used			Collection year	Province
	Intestines	Muscle	Spleen		
Raccoon 189	SVT	n.a.	n.a.	2010	Salzburg
Raccoon 229	SVT	n.a.	n.a.	2010	Lower Austria
Raccoon 251	SVT	n.a.	n.a.	2011	Styria
Raccoon 253	SVT	n.a.	n.a.	2010	Styria
Raccoon 311	n.a.	n.a.	PCR	2013	Lower Austria
Raccoon 328	SVT	n.a.	PCR	2015	Carinthia
Raccoon 400	SVT	Diaphragm (<5 g)	PCR	2015	Lower Austria
Raccoon 401	SVT	Diaphragm	PCR	2016	Lower Austria
Raccoon dog 211	SVT	n.a.	n.a.	2010	Burgenland
Raccoon dog 247	SVT	n.a.	n.a.	2011	Burgenland
Raccoon dog 255	SVT	Limb	n.a.	2011	Burgenland
Raccoon dog 256	SVT	Limb (<5 g)	n.a.	2011	Lower Austria
Raccoon dog 266	SVT	Limb	n.a.	2011	Lower Austria
Raccoon dog 293	SVT	n.a.	PCR	2012	Burgenland
Raccoon dog 314	SVT	n.a.	PCR	2014	Vorarlberg
Raccoon dog 321	n.a.	n.a.	PCR	2014	Lower Austria
Raccoon dog 322	n.a.	n.a.	PCR	2014	Lower Austria
Raccoon dog 324	n.a.	n.a.	PCR	2014	Lower Austria
Raccoon dog 389	SVT	Diaphragm	PCR	2015	Lower Austria
Raccoon dog 394	SVT	Diaphragm	PCR	2015	Styria
Raccoon dog 397	SVT	Limb/Diaphragm	PCR	2015	Lower Austria

*PCR* polymerase chain reaction used for piroplasmids and Anaplasmatacae, *SVT* shaking in a vessel technique, *n.a*. not available

## Results

Concerning the human relevant parasites, we detected *A. alata* in the intestines of three raccoon dogs (30%) as well as *E*. *multilocularis* in one raccoon dog intestine (10%) (Table 2). The examined raccoon dogs were also infected with *Uncinaria stenocephala* (40%), *Mesocestoides* spp. (40%), *Molineus* spp. (30%), *Toxocara canis* (20%), *Taenia* spp. (20%), *Isthmiophora melis* (20%), *Dipylidium caninum* (10%) and *Toxascaris leonina* (10%). Seven examined raccoons were free of gastrointestinal parasites, and one specimen was infected with *Molineus* spp.

**Table 2:** Parasite species detected in the examined raccoon (left) and raccoon dog (right) specimens and infestation intensity (middle intense for *Echinococcus multilocularis*: 101–1000 specimens, low intense for *Mesocestoides* sp.: below 30 specimens)

Species	Raccoon								Raccoon dog												
ID	189	229	251	253	311	328	400	401	211	247	255	256	266	293	314	321	322	324	389	394	397
*Alaria alata*	0	0	0	0	–	0	0	0	6	0	23	0	37	0	0	–	–	–	0	0	0
*Isthmiophora melis*	0	0	0	0	–	0	0	0	0	0	9	1	0	0	0	–	–	–	0	0	0
*Dipylidium caninum*	0	0	0	0	–	0	0	0	0	0	0	0	0	0	0	–	–	–	0	0	1
*Echinococcus multilocularis*	0	0	0	0	–	0	0	0	0	0	0	0	0	0	Middle intense	–	–	–	0	0	0
Mesocestoides sp.	0	0	0	0	–	0	0	0	0	1	1	0	0	Low intense	5	–	–	–	0	0	0
Taenia sp.	0	0	0	0	–	0	0	0	0	0	0	0	0	0	0	–	–	–	1	0	3
Molineus sp.	1	0	0	0	–	0	0	0	1	12	1	0	0	0	0	–	–	–	0	0	0
*Toxascaris leonina*	0	0	0	0	–	0	0	0	0	0	0	1	0	0	0	–	–	–	0	0	0
*Toxocara canis*	0	0	0	0	–	0	0	0	0	0	0	1	0	0	0	–	–	–	0	0	5
*Trichinella* sp.	–	–	–	–	–	–	0^a^	0	–	–	0	0^a^	0	–	–	–	–	–	0	0	0
*Uncinaria stenocephala*																					
*Babesia* cf. *microti*	–	–	–	–	0	0	0	0	–	–	–	–	–	1	1	0	1	0	1	0	1
Anaplasmatacae sp.	–	–	–	–	0	0	0	0	–	–	–	–	–	0	0	0	0	0	0	0	0

^a^Less than 5 g of muscle available

The results of the PCR showed no Anaplasmataceae infections of the four raccoons as well as of the eight raccoon dogs examined. However, none of the raccoons but five of eight raccoon dogs were tested positive for *B*. cf. *microti* (Genbank® accession number [KY246306]). The sequence (partial 18S rRNA) were 100% identical to each other and to the sequences found in foxes from Austria (e.g. [KM115972]). The *Trichinella* analysis showed all of the investigated raccoon and raccoon dog samples were free of *Trichinella* spp.—although, two of the samples consisted of less than 5 g and thus were probably not representative.

**Fig. 1:**
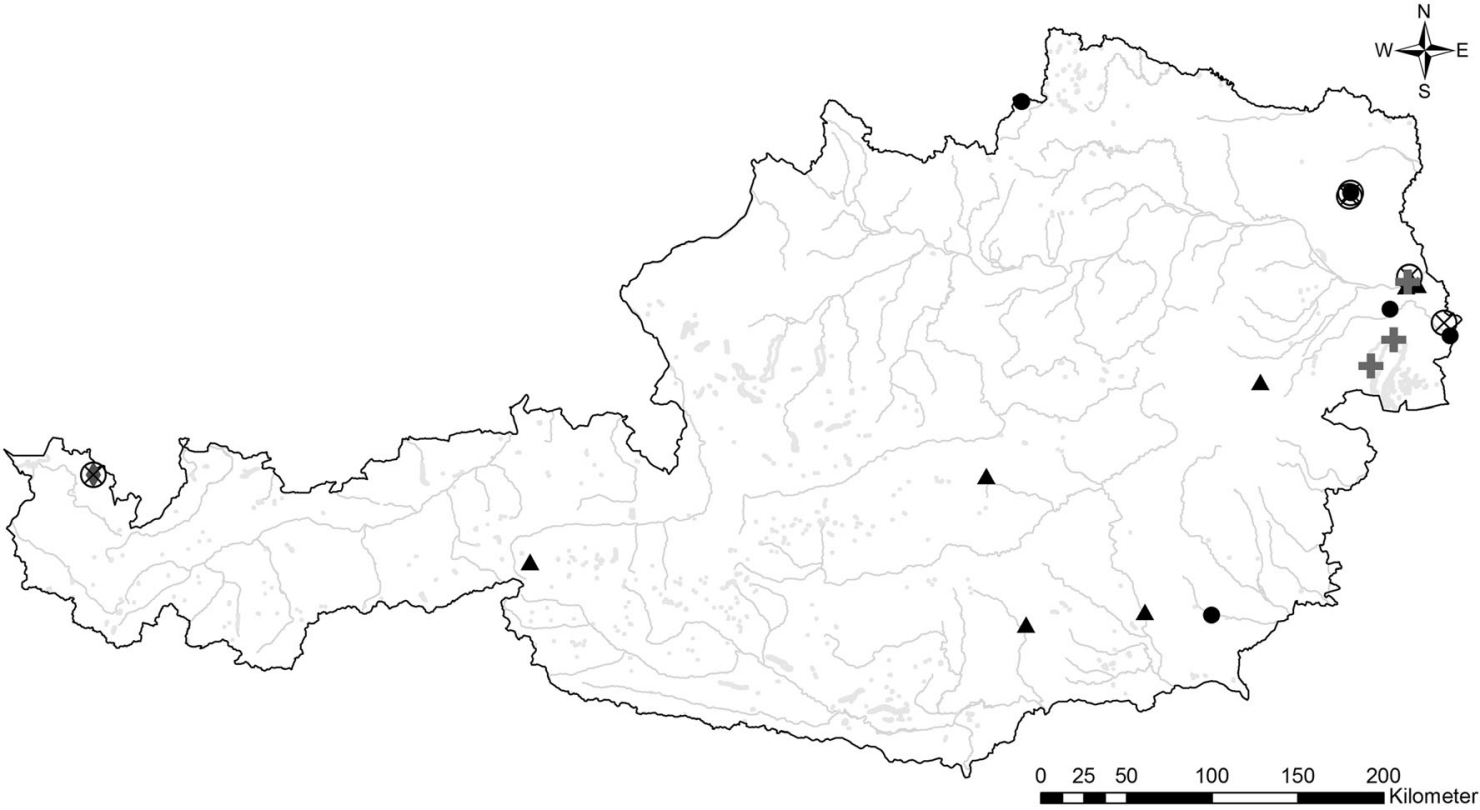
Geographical distribution of raccoon dogs infected with *Alaria alata* (), *Babesia* cf. *microti* () and *Echinococcus multilocularis* () as well as of raccoons (▲) and raccoon dogs (•) not infected with the relevant parasites (see above)

The *A. alata* infected raccoon dogs originated from eastern Austria, precisely from the Lake Neusiedl and the Danube floodplain areas (Fig. 1), while the *E*. *multilocularis* positive raccoon dog was shot in the northwestern edge of the country. Tissues of the racoon dogs infested with *B.* cf. *microti* were sampled in the east as well as in the west.

## Discussion

Interestingly, the *E*. *multilocularis* appeared in the western areas of Austria, those areas of known high endemicity of this cestode in foxes (Duscher et al. 2006). The host competence of the raccoon dog for *E*. *multilocularis* is in accordance with studies from other countries (e.g. Al-Sabi et al. 2013; Laurimaa et al. 2015; Schwarz et al. 2011; Thiess et al. 2001). But, due to its feeding habits, the raccoon dog seems to play a minor role as a reservoir of *E*. *multilocularis* than the red fox does (Al-Sabi et al. 2013; Bružinskaitė-Schmidhalter et al. 2012).

In contrast, the trematode *A. alata* was found in the eastern parts surrounding the Lake Neusiedl, and in the Danube floodplains where this parasite was also found in foxes (Duscher 2011), and where the densities of paratenic hosts are high. These wetlands are also the areas with a high probability of raccoon dog presence (Duscher and Nopp-Mayr under review), and where future population densities are expected to be high. Our results confirm previous European studies that showed a high abundance of *A*. *alata* in raccoon dogs and thus a probable high susceptibility of the raccoon dog for this gastrointestinal parasite with high zoonotic relevance (Al-Sabi et al. 2013).

Furthermore, *B*. cf. *microti* was found in five out of eight investigated raccoon dogs. To our knowledge, this is the first evidence of a *B*. cf. *microti* infection of raccoon dogs in Europe and is in accordance with the idea of sharing the fox parasites, as seen for the gastrointestinal parasites. In this context, the raccoon dog may be seen as additional reservoir of *B*. cf. *microti* as already suggested by Han et al. (2010). Contrary to that, the raccoons were negative for this pathogen, although the *B*. cf. *microti* species found in Austrian foxes is closely related to those found in raccoons in Japan (Baneth et al. 2015). So, in this case, the same type of pathogen also could circulate among the host species. Unfortunately, the sample size is too low to either confirm or reject this assumption.

No Anaplasmataceae were found, which might have occurred in the raccoons, e.g. the raccoon-associated bacteria *Candidatus* Neoehrlichia lotoris (CNL) as stated in previous works (Hodžić et al. 2015, 2016). These bacteria were found and described in raccoons from the USA (Yabsley et al. 2008), but recently were accidentally found in foxes from Austria and the Czech Republic (Hodžić et al. 2015, 2016), far away from the US distribution. Likewise, all examined raccoon and raccoon dog samples were free of *Trichinella* spp., but due to the small sample size, no clear declaration can be made.

Generally, our examined raccoons were almost pathogen free, which also was observed in other studies within their introduced range (e.g. Schwarz et al. 2015). The idea is that in some regions, introduced raccoons were originating from fur farms and dewormed in regular manner. Thus, geographical differences in the worm prevalence, e.g. *B*. *procyonis*, within their introduced range are supposed to depend on the source population (Schwarz et al. 2015; Winter et al. 2005). In opposite to the raccoon dog, the raccoon is not that suitable for fox parasites (Schwarz et al. 2015; Thiess et al. 2001). This might be the explanation for the overall lower prevalence in the investigated raccoons in this study.

## Conclusion

Due to the low population densities of the raccoon and the raccoon dog in Austria (Duscher 2016), our sample size is quite low and varying. Nevertheless, our results support the thesis of Michler and Michler (2012) that the epidemiological meaning of the raccoon in Europe is still low. However, a further spread and population increase of this non-indigenous species will obviously lead to an admixture of the different founder populations (Biedrzycka et al. 2014; Fischer et al. 2015), and therewith to a further spread of *B*. *procyonoides* as well*.* In the case of the raccoon dog, our results confirm it as a host of *E*. *multilocularis* as well as a probable indicator species of *A*. *alata*. As the origins of *A. alata* positive samples are also the regions with expected high raccoon dog population densities (Duscher and Nopp-Mayr under review), *A. alata* infections is an increasing risk. Moreover, our study confirms the raccoon dog as an additional host of *B*. cf. *microti* with a relevant impact as a reservoir. Concerning its epidemiological impact and facing a further spread in Europe, the raccoon dog should be monitored more intensively in the matters of vector-borne diseases (Sutor et al. 2014).
